# Molecular Signatures of Blood Biomarkers in Depression: Gene Expression Analysis of *GAR1, PER3, MTPAP, SLC25A26*, and *CD19*, a Case–Control Study

**DOI:** 10.3390/diagnostics16060884

**Published:** 2026-03-16

**Authors:** Remya Bhaskaran S, Ramya Sugumar, Suvarna Jyothi Kantipudi, C. D. Mohana Priya, C. Sheela Sasikumar

**Affiliations:** 1 Department of Pharmacology, Sri Ramachandra Institute of Higher Education and Research (SRIHER), Porur, Chennai 600116, India; remyabhaskarans@sriramachandra.edu.in; 2 Department of Psychiatry, Sri Ramachandra Institute of Higher Education and Research (SRIHER), Porur, Chennai 600116, India; 3 Department of Human Genetics, Sri Ramachandra Institute of Higher Education and Research (SRIHER), Porur, Chennai 600116, India; 4 Dr. RK Diabetic Foot and Podiatric Institute & Rakesh Jhunjhunwala Amputation Prevention Centre, Chennai 600099, India

**Keywords:** mental health, major depressive disorder, mental wellness, gene expression, qRT-PCR, CD19, MTPAP, PER3, biomarkers, ROC analysis, peripheral blood, psychiatric diagnosis

## Abstract

**Background/Objective:** Major depressive disorder (MDD) is a common mental illness that lacks objective diagnostic biomarkers and has a complicated pathogenesis. Peripheral blood-based gene expression profiling provides a potential, non-invasive method to identify the molecular markers associated with risk stratification and depression severity.This study aimed to investigate the gene expression of five candidate genes—*CD19*, *MTPAP*, *PER3*, *GAR1*, and *SLC25A26*—in the peripheral blood of drug-naïve patients with MDD and to evaluate their potential as diagnostic biomarkers. **Methods:** Peripheral blood samples were collected from 100 newly diagnosed, drug-naïve MDD patients and 100 age- and sex-matched healthy controls. The total RNA was extracted and reverse-transcribed for quantitative real-time PCR analysis. Fold change and ∆∆Ct values were calculated for each gene, followed by statistical analysis using *t*-tests and ANOVA. Receiver operating characteristic (ROC) curves were used to evaluate diagnostic performance. Gene co-expression and pathway enrichment analyses were conducted to explore functional relevance. **Results:** Significant upregulation was observed for CD19 (fold change = 6.15, *p* = 6.17 × 10^−10^), MTPAP (fold change = 3.99, *p* = 1.74 × 10^−8^), and PER3 (fold change = 2.42, *p* = 5.33 × 10^−6^) in MDD. GAR1 showed modest upregulation (fold change = 1.26, *p* = 0.47), while SLC25A26 (fold change = 1.24, *p* = 0.47) was not significantly altered. Combined ROC analysis yielded an AUC of 0.885, indicating a strong discriminative ability. Gene expression correlated with depression severity (HAM-D and PHQ-9). **Conclusions:** This study identifies *CD19*, *MTPAP*, and *PER3* as promising peripheral blood biomarkers for MDD, with potential implications for early diagnosis, severity assessment, and personalized treatment strategies.

## 1. Introduction

The World Health Organization (WHO) reports that major depressive disorder (MDD) impacts more than 280 million individuals globally, and is a common and debilitating mental illness that is one of the most important factors influencing the worldwide burden of disease and disability [1,2,3,4]. It is characterized by neurovegetative symptoms, cognitive deterioration, anhedonia, fatigue, and chronic depression with suicidal thoughts or attempts [5,6]. Around 10% of pregnant women and postpartum mothers are affected by depressive symptoms, and the disease is almost 50% more common in women than in males [1,7]. Among those aged 15 to 29, suicide ranks as the fourth most frequent cause of death, with over 700,000 deaths reported annually [1]. Depressive disorders not only reduce the quality of life but also increase the risk of concomitant diseases such as diabetes, heart disease, and immunological dysfunction. Depression is still mostly diagnosed by subjective clinical evaluations based on symptomatology, despite its significant prevalence worldwide [7]. Misdiagnosis, delayed therapy initiation, and inconsistent therapeutic results are frequently caused by this subjectivity. Even though MDD is quite common and has a significant socioeconomic impact, it is still challenging to identify the illness promptly and offer individualized treatment, since the underlying biological pathways are still poorly understood [8]. Since there are no objective biological markers for depression, there is growing interest in finding peripheral biomarkers, especially in readily available tissues like blood, to help with the diagnosis, risk assessment, and monitoring of therapeutic responses [9]. Biomarkers can predict the therapeutic response to antidepressants, predict illness vulnerability, and identify patients who are more likely to be suicidal or resistant to treatment [8,9,10,11]. High-throughput transcriptomic technologies have made it possible to identify differentially expressed genes (DEGs) in peripheral blood. These DEGs may be indicative of pathophysiological processes related to MDD, such as immune dysregulation, mitochondrial dysfunction, epigenetic changes, and circadian rhythm disruption [12,13,14,15]. These processes may aid in bridging the gap between neurobiology and clinical presentation, as they have been frequently linked to the pathophysiology of depression. In this regard, transcriptome profiling studies of MDD patients, especially those examining the suicide risk and neurobiological subtypes of depression, have produced a number of gene candidates [6,16,17,18,19]. Prominently, blood samples from people with depression have demonstrated different expression of genes like *GAR1* (involved in ribonucleoprotein biogenesis), PER3 (a crucial circadian clock regulator), MTPAP (a mitochondrial polyadenylation factor), SLC25A26 (a mitochondrial SAM transporter), and CD19 (a B-lymphocyte surface marker) when compared to the healthy controls [20,21,22,23]. Notably, the pathophysiology of mood disorders has been extensively associated with genes related to circadian rhythms, specifically PER3. Changes in PER3 have an impact on affective stability, cognitive function, and sleep–wake regulation. According to recent genetic research by Barlattani et al. (2024) [24],PER3 variable number tandem repeat (VNTR) mutations influence the age of onset and the clinical course of bipolar I disorder, highlighting the crucial function of the gene in mood phenotypic modulation and circadian disruption [24,25]. Incorporating these circadian genetic insights offers a solid scientific basis for investigating PER3 expression in depression, where sleep and rhythm abnormalities are important clinical characteristics. Predicting the changes in gene expression in peripheral blood may therefore help with early diagnosis and open the door for precision medicine techniques in psychiatry [26,27]. In this study, the peripheral blood samples of newly diagnosed, drug-naïve patients with depression were examined for the expression levels of five identified candidate genes: *GAR1*, *PER3*, *MTPAP*, *SLC25A26*, and *CD19*. The results were compared with those of age- and sex-matched healthy controls. Additionally, we investigated their potential use as diagnostic biomarkers using receiver operating characteristic (ROC) analysis and looked at potential gene–gene relationships, using enrichment and co-expression network studies. This study aims to build objective diagnostic tools for clinical psychiatry and improve our understanding of peripheral molecular biomarkers in depression by combining molecular discoveries with clinical characteristics.

## 2. Methodology

A committee dedicated to institutional ethics at Sri Ramachandra Institute of Higher Education and Research (SRIHER), Chennai, validated and approved this research (Ref: IEC-NI/23/MAY/87/23). All procedures involving human participants were performed in accordance with the relevant guidelines and regulations. This is a diagnostic accuracy study that followed the STARD (Standards for Reporting Diagnostic Accuracy Studies) 2015 guidelines to ensure transparency in reporting the participant recruitment, study methodology and the statistical analysis, provided as Supplementary Material. This study comprised 200 participants in total, divided into two groups: a case group of 100 newly diagnosed patients with depression and a healthy control group of 100 persons who were matched for age and sex. Based on previous research assessing differential gene expression in depression and a power calculation that aimed for an effect size of 0.5 with 80% power and a significance level of 0.05, the sample size was established, ensuring appropriate statistical reliability for multigene comparisons. The period from January 2023 to July 2024 was used for data collection, and the patients with newly diagnosed depression were recruited from the Sri Ramachandra Medical Centre outpatient psychiatry department after obtaining written informed consent. This study included both case and control groups for ages ranging from 18 to 45 years. The DSM-5 criteria were used to diagnose depression, and the severity was assessed using the validated, standardized clinical scales—the Hamilton Depression Rating Scale (HAM-D) and the Patient Health Questionnaire-9 (PHQ-9). Based on their scores, 91 individuals in the case group were diagnosed as having severe depression, and 9 patients were identified as having moderately severe depression out of the 100 patients. Peripheral blood samples (*n* = 200) were collected from both the case and control groups in an EDTA-coated vacutainers for the molecular analysis. Using the standardized protocol, the obtained blood samples were processed right away or kept at 4 °C until the RNA was extracted for quantitative gene expression analysis, with an emphasis on the expression levels of chosen candidate genes.

### 2.1. Isolation of Ribonucleic Acid (RNA)

The whole blood samples collected in an EDTA vacutainer were used for the isolation of total RNA by the TRIzol reagent (Invitrogen) method. This is done after the separation of lymphocytes by the Ficoll density gradient centrifugation. To obtain and isolate the buffy coat, 1 μL of whole blood samples were carefully layered along the sides of the tube and centrifuged for 45 min at 4000 rpm using 1 μL of Ficoll. The resultant obtained as a buffy coat was then transferred to a new tube and 500 μL 1× phosphate-buffered saline (PBS) was added, and it was centrifuged for 15 min at 10,000 rpm [28]. The pellets obtained were lysed in TRIzol reagent and incubated for 10 min. After adding 200 μL of chloroform to this mixture, it was centrifuged for ten min. After collecting the resulting supernatant, 500 μL of isopropyl alcohol was added, and the mixture was centrifuged for 10 min at 10,000 rpm. The resulting supernatant was then disposed of, and the RNA pellets were left to air dry. For future use, the resultant RNA pellets were stored at −80 °C after being dissolved in RNAse-free water.

### 2.2. RNA Quantification and cDNA Conversion

A nanodrop spectrophotometer was used at an absorbance ratio of 260/280 nm for the assessment of the purity and quantification of the isolated RNA. For the synthesis of cDNA, the High-Capacity cDNA Reverse Transcription Kit (Applied Biosystems) was used to reverse-transcribe 1 μg of the total RNA in a 20 μL reaction volume. In a thermal cycler, the reverse transcription process was run under a series of temperature settings: 25 °C for 10 min, 37 °C for 120 min, 85 °C for 5 min, and then held at 4 °C.

### 2.3. qRT-PCR (Quantitative Real-Time PCR)

Following reverse transcription, the cDNA serves as a template for the quantification of the gene expression profile, using qRT-PCR for all the five selected candidate genes in depression: *GAR1*, *PER3*, *MTPAP*, *SLC25A26*, and *CD19*. Table 1 lists the particular forward and reverse primers for these chosen genes. The SYBR Green Master Mix (Qiagen), primers for both directions, ROX dye, and RNase-free water, along with cDNA templates were used in the amplification procedures. The Rotor-Gene Q system (Qiagen, Germany) was used for each analysis. After loading the reaction tubes into the qRT-PCR apparatus, the tubes were first denatured at 95 °C for five minutes. This was followed by 40 cycles of denaturation at 95 °C for ten seconds, annealing at 60 °C for ten seconds, and an extension at 72 °C for thirty seconds. The endogenous control for normalization was β-actin, and the amplification specificity was assessed using a melt curve analysis.

#### Statistical Analysis

The 2^(−ΔΔCt) approach was used to evaluate the gene expression levels, where ΔCt = Ct (target gene) − Ct (reference gene) and ΔΔCt = ΔCt (depression group) − ΔCt (control group). Therefore, when comparing MDD to controls, a fold change greater than one signifies upregulation, while a fold change of less than one implies downregulation. To enhance the interpretative transparency, the same directionality convention was used consistently throughout the work, and all fold-change values are displayed on a linear (non-logarithmic) scale.

IBM SPSS Statistics v26.0 and GraphPad Prism 10 were used to examine the data. Gene expression between two groups was compared using Student’s *t*-test, and demographic characteristics were analyzed using a one-way ANOVA. The normality of the data was analyzed using the Shapiro–Wilk test. Using the Bonferroni correction, statistical significance was defined at *p* < 0.01 to account for the multiple testing of five genes. Diagnostic accuracy was evaluated using receiver operating characteristic (ROC) analysis.

## 3. Result

### 3.1. Demographic Analysis

The principal aim of this investigation was to identify a possible blood biomarker for depression by using the gene expression profile of both the patients and the healthy controls. Table 2 shows the demographic details of both the patient and the control group. The control group’s mean age was 32.3 ± 8.3 years (males: 55, females: 45), and in the depression group, it was 32.7 ± 7.3 years (males: 57, females: 43). No significant difference in age or sex was observed between the groups. ANOVA was done to determine the statistical significance of the differences in the expression. The GraphPad Prism software was used for all the graphs and statistical analyses.

“Age was compared using the independent sample *t*-test, Chi-square test was used for the analysis of gender distribution.”

### 3.2. Gene Expression Analysis

The expression levels of the five chosen genes were compared between both the patients and the control groups, using the quantitative RT-PCR of whole blood samples. “The **cycle threshold (Ct)** value is inversely proportional to the targeted nucleic acid concentration, signifying that a few cycles are needed for the fluorescent signal to surpass the threshold, and hence used for the assessment of gene expression analysis. A low ct value signifies the higher gene expression” [29]. The Ct value for each sample was determined using the difference calculated between the targeted gene and the housekeeping gene (*β-actin*). The average Ct value was obtained for both the patient and control group, using the following formula:

“∆∆Ct = ∆Ct of target gene − ∆Ct of Control”.

Additionally, each gene’s fold change was computed using the formula 2^(−∆∆Ct). A fold change value greater than one signifies upregulation, while a fold change less than one implies downregulation.

**Figure 1:**
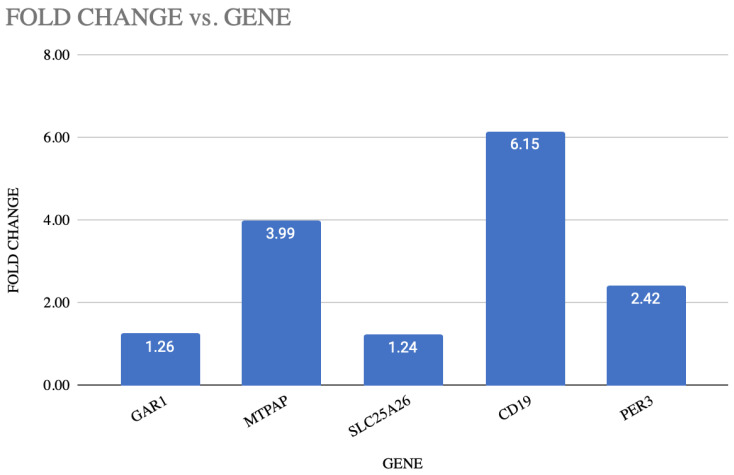
Illustration of fold change and *p*-value of each gene.

**Figure 2:**
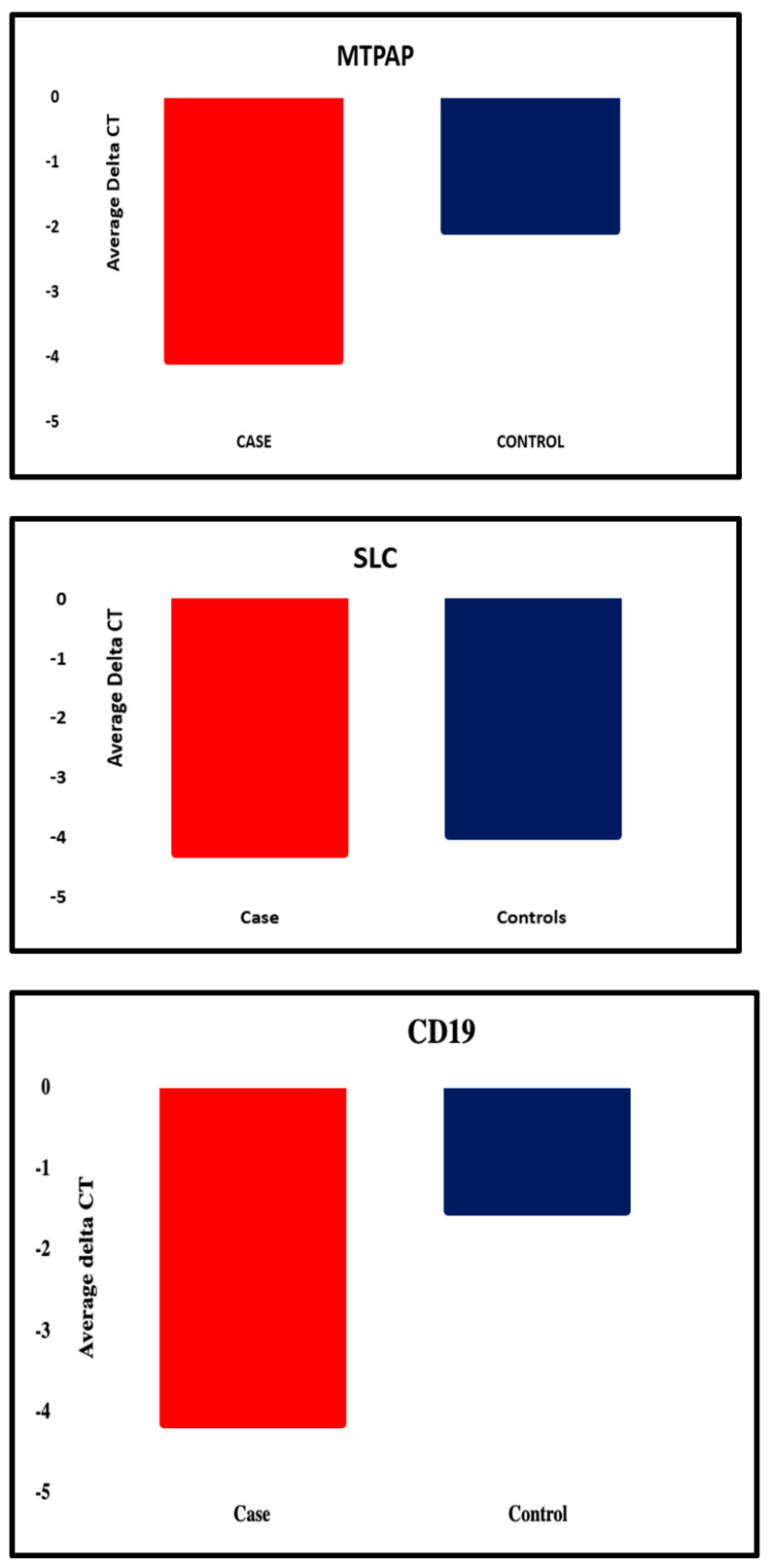

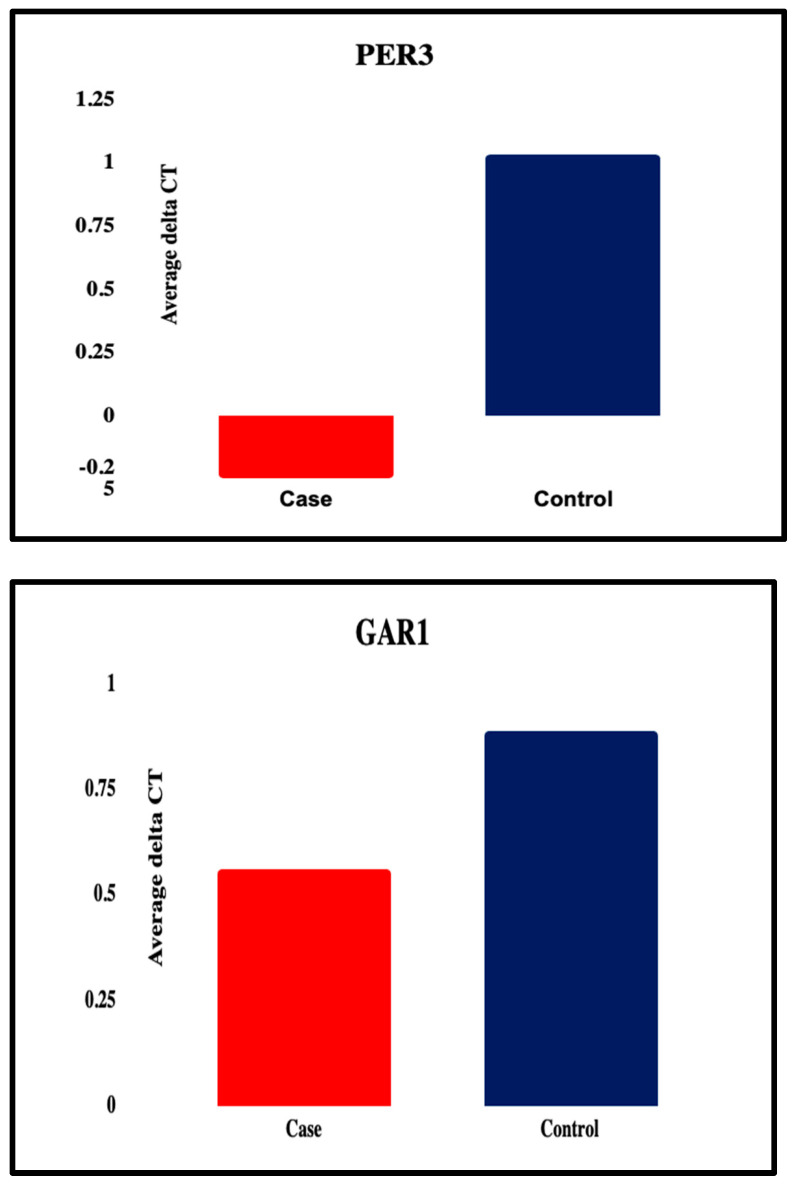
Graphical representation of all the five candidate genes that were significantly differentially expressed between the patient and the control group.

The SPSS program, as well as GraphPad Prism 10, was used to analyze the data. To assess the normality of the data, the Shapiro–Wilk test was employed, and Student’s *t*-test was used to compare groups. In order to reduce the Type I error, the Bonferroni correction was used with a reduced significance level of *p* < 0.01 in light of the five genes that were analyzed. The analysis revealed that three of the five genes were significantly and statistically upregulated in depression. A strong upregulation was shown by the *CD19* gene, with a *p*-value of 6.17 × 10^−10^ and fold change of 6.15 in depression, followed by MTPAP, with a *p*-value of 1.74 × 10^−8^ and fold change of 3.99. The *p*-value of 5.33 × 10^−6^ and fold change of 2.42 shows the upregulation of PER3 in depression. This shows the potential role of PER3 in the molecular mechanism of depression. The *p*-value of 0.48 and a fold change of about 1.24 indicate that SLC25A26 and GAR1 with a *p*-value of 0.47 and fold change of 1.26 showed the modest changes in gene expression. These findings imply that mitochondrial activity (*MTPAP*), immunological response (*CD19*), and circadian rhythm (*PER3*) genes have altered transcriptional regulation in depressed patients. Table 3 shows the differential expression of blood-based genes, along with the fold change between MDD patients and the control group. Figure 1 represents the fold change in each gene with the *p*-value. Figure 2 illustrates the graphical representation of all the five candidate’s genes that are significantly expressed between the patient and the control in blood.

### 3.3. Bar Diagram

Using the fold change in each of the five genes’ gene expression analysis, the mean fold change and the standard deviation were calculated for both the case and the control group. These values were used to create a bar diagram in GraphPad Prism 10 that demonstrated that the mean and standard deviation of the *GAR1* gene in depression (3.58 ± 3.29) showed a moderate upregulation compared to those in the control group (2.94 ± 0.54).

**Figure 3:**
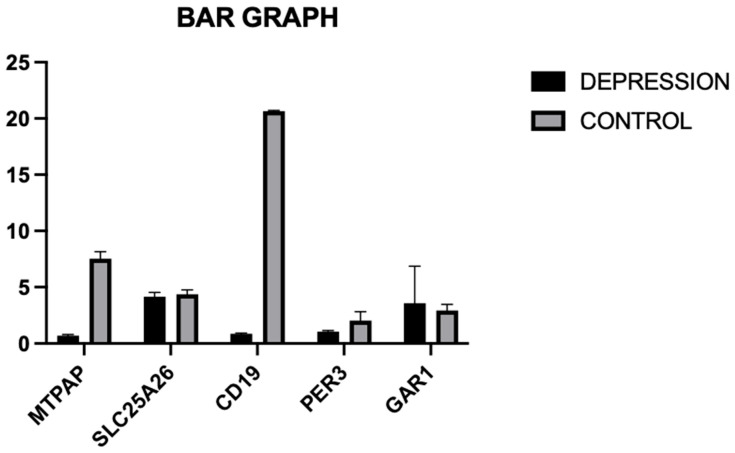
Illustration of fold change in each gene, using a bar diagram for both the case and the control group.

Among the five selected genes (***GAR1***, ***PER3***, ***MTPAP***, ***SLC25A26***, and ***CD19***), only one gene, *GAR1*, showed moderately significant upregulation, while ***PER3***, ***MTPAP***, ***SLC25A26***, and ***CD19*** were significantly upregulated in the patients’ group when compared to the healthy controls with fold change value (PER3: 2.42, CD19: 6.15, MTPAP: 3.99, SLC25A26: 1.24). This differential upregulation in gene expression suggests the potential role of these genes in the pathophysiology of depression and is illustrated in Figure 3.

### 3.4. Receiver Operating Characteristic (ROC) Analysis

To determine the potential blood-based biomarker for depression, ROC analysis was performed, using the expression of all the five genes. ROC analysis is the most commonly used graphical tool in medical decision making that is particularly useful for evaluating the effectiveness of various diagnostic tests [30]. It plots the sensitivity (true positive rate) against one specificity (false positive rate) at different threshold values. The test’s capacity to distinguish between the patients and control group across all potential thresholds is summed up by the area under the curve (AUC).

**Figure 4:**
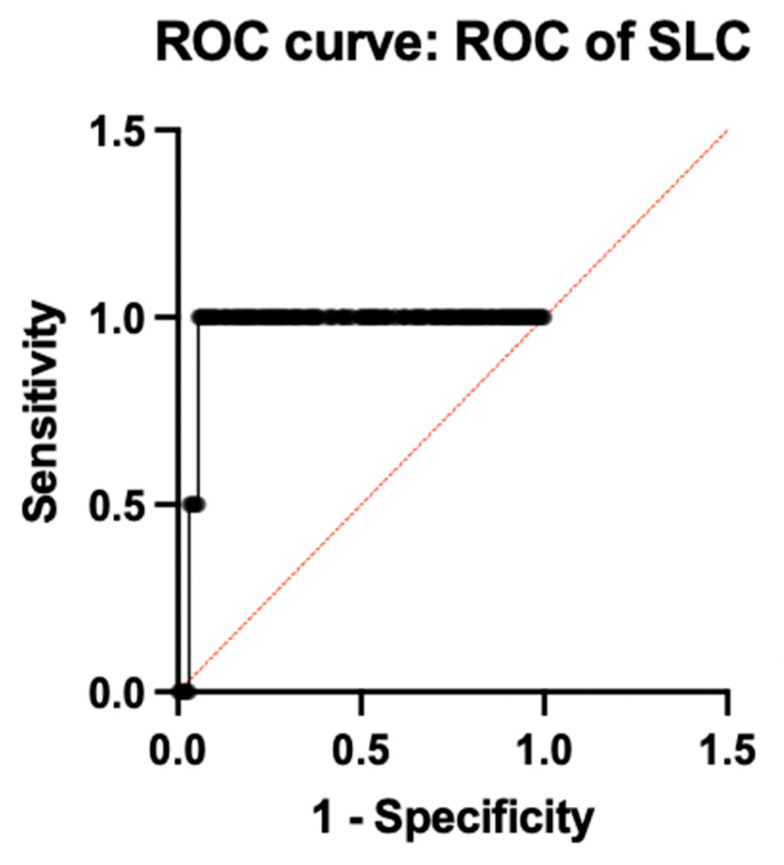

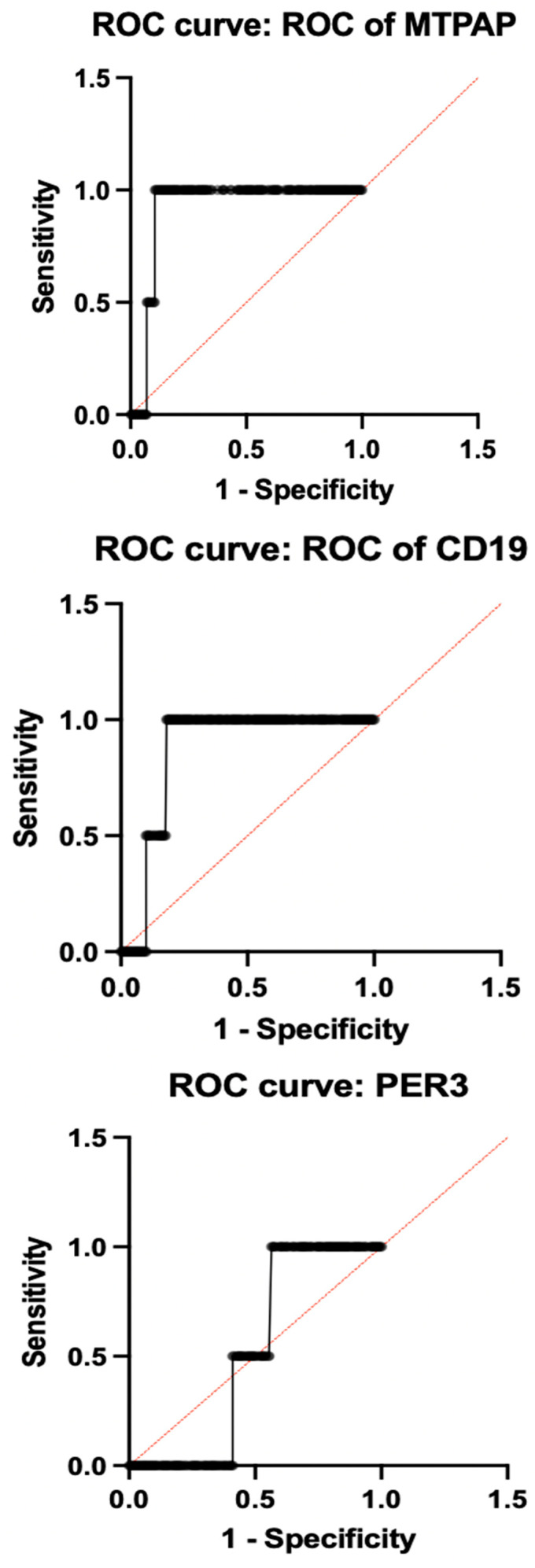

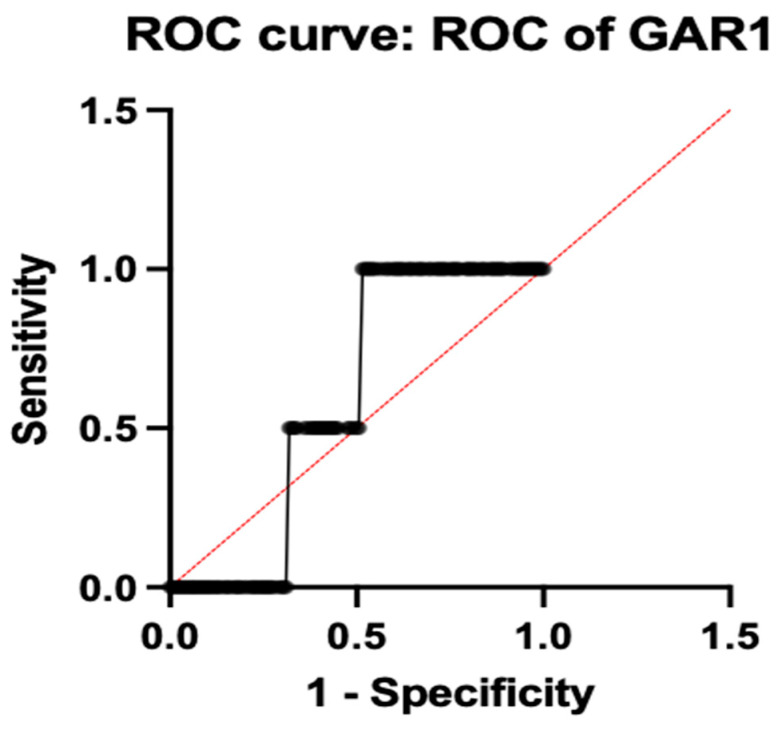
ROC analysis of individual genes.

Among the individual genes with a sensitivity of 98.1% and a specificity of 91.2% with an ideal ΔCt cut-off of −4.33, as determined by Youden’s index, SLC25A26 had the highest diagnostic accuracy (AUC = 0.9575; 95% CI: 0.93–10.98). Additionally, MTPAP demonstrated outstanding performance (AUC = 0.9125; 95% CI: 0.88–0.94), while CD19 followed closely behind (AUC = 0.8613; 95% CI: 0.82–0.90), indicating their possible function as potent biomarkers associated with the immune system and mitochondria, respectively. Both PER3 (AUC = 0.5150; 95% CI: 0.45–0.57) and GAR1 (AUC = 0.5875; 95% CI: 0.52–0.65) showed low discriminatory power, suggesting only a minor or non-specific diagnostic contribution. Figure 4 illustrates the ROC of individual genes. All the accuracy measures, such as sensitivity, specificity, AUC, and cross-validation, were reported based on the STARD 2015 guidelines.

**Figure 5:**
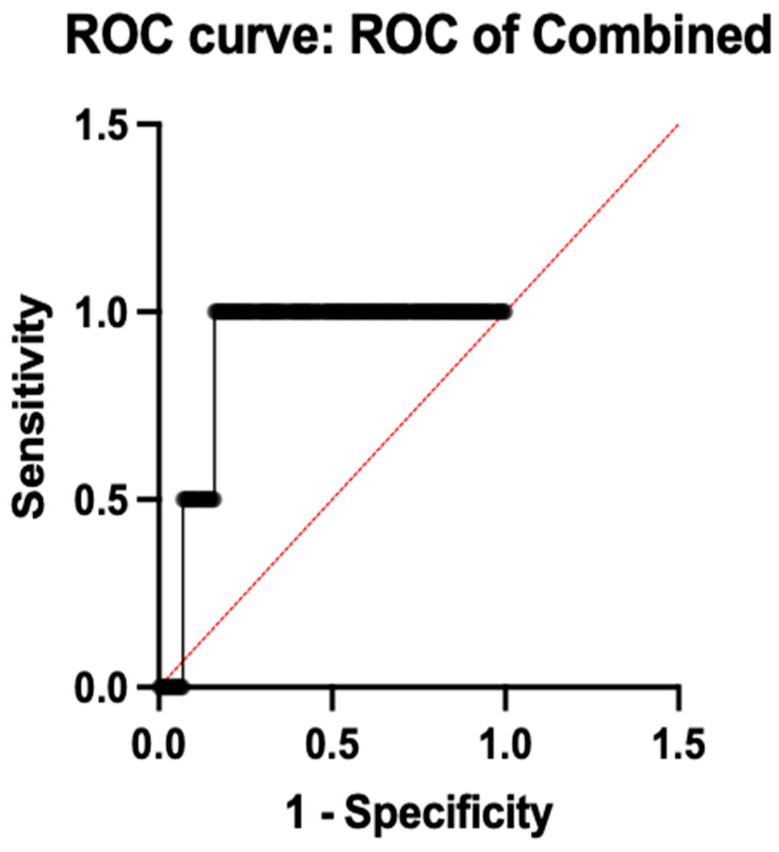
ROC analysis of combined genes.

To evaluate the diagnostic performance of combined gene expression, ROC analysis was done, which revealed an AUC of 0.8850 (95% CI: 0.85–0.92; *p* < 0.0001), 97.5% sensitivity, and 82.0% specificity, demonstrating good diagnostic capacity when all five genes were combined into a multi-marker model. A 10-fold cross-validation was used to evaluate the trustworthiness of these results, and the mean cross-validated AUC of 0.876 ± 0.019 confirmed the model stability and reduced over-fitting. Table 4 shows the comprehensive diagnostic performance metrics for each gene and the combined model. This is illustrated in Figure 5.

### 3.5. Gene Co-Expression Analysis

**Figure 6:**
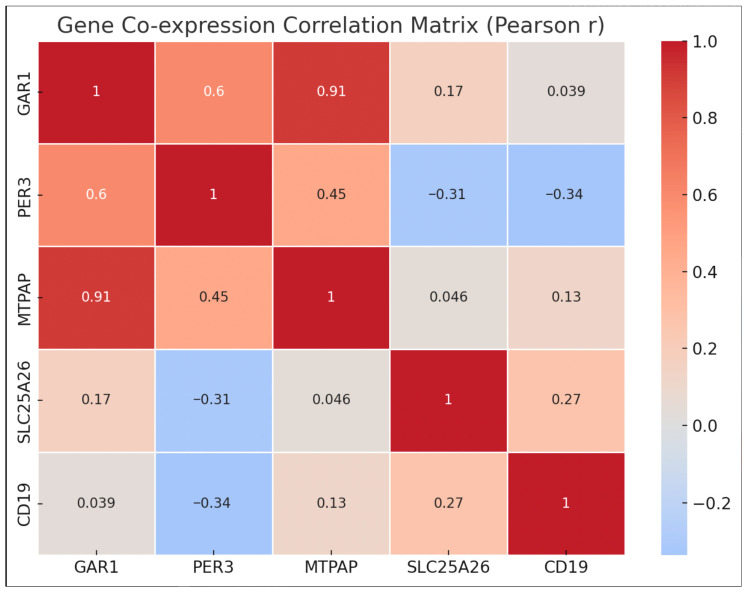
Co-expression network diagram.

To determine the correlation between the five genes that are expressed differently in depressed patients and healthy controls, a bio-informatics approach to gene co-expression analysis was performed using the gene expression profile of *GAR1*, *PER3*, *MTPAP*, *SLC25A26*, and *CD19* [31]. A pairwise Pearson correlation coefficient (r) analysis was performed, using the ∆Ct values of these genes to explore the significance of correlations in their pattern of gene expression, and to understand its involvement in any of the biological processes. No correlation was observed with statistical significance (*p* < 0.05) among these genes, which revealed a lack of co-regulation. This is illustrated in Figure 6.

**Figure 7:**
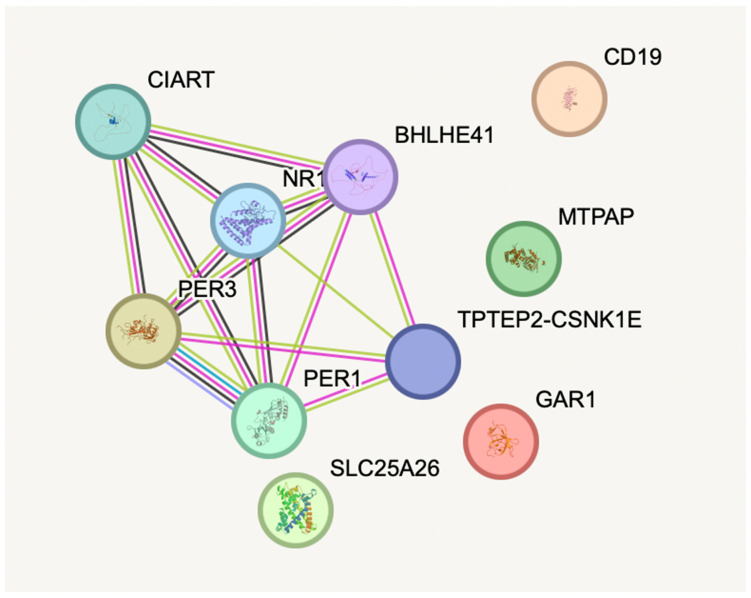
Protein–protein interaction and functional enrichment analysis using the STRING database.

**Network and pathway enrichment analysis** was done for all of the five genes: *GAR1*, *PER3*, *MTPAP*, *SLC25A26*, and *CD19*, that are differentially expressed in depression. Figure 7 shows the protein–protein interaction and functional enrichment analyses using the STRING database and showed no significant enrichment, but helped to reveal certain associated pathways using the biologically realistic annotations. [32,33] *GAR1* was associated with ribosome biogenesis and pseudo uridylation of rRNA, highlighting its important role in the regulation of translation. A plausible association was found between PER3 and circadian rhythm pathways, confirming the evidence for the depressive disorder. Both MTPAP and SLC25A26 were linked to mitochondrial RNA processing and SAM transport, supporting the impaired mitochondrial function and methylation metabolism, as potential causes of neuropsychiatric symptoms. CD19 was associated with the B-cell receptor signaling, and immune dysregulation in depression. [14,34] Thus, these findings show the significant need for future studies focusing on functional enrichment and validation.

### 3.6. Clinical Correlation of Gene Expression with Depression Severity

This study also included the severity of depressive symptoms, as measured by standardized clinical scales—the **Patient Health Questionnaire-9 (PHQ-9)** and the **Hamilton Depression Rating Scale (HAM-D)**—among the patients group compared to the healthy controls, and correlated the severity with the gene expression of all of the five differentially expressed genes: *CD19*, *MTPAP*, *PER3*, *SLC25A26*, and *GAR1*. In this study, a biological connection between disease severity and molecular dysregulation may be shown by the significantly increased expression of CD19, MTPAP, and PER3 in individuals with higher scores in these measures, which are suggestive of moderately severe to severe depression. The significant upregulation of CD19 (fold change 6.15, *p* = 6.17 × 10^−10^) in those with high HAM-D and PHQ-9 scores supports the idea that immunological dysregulation plays a major role in severely depressed episodes. Similarly, MTPAP and PER3 are also significantly upregulated, which is associated with the disturbances in circadian rhythm regulation and mitochondrial energy metabolism. These processes are frequently compromised in patients who have higher levels of depressive symptoms. On the other hand, there were no significant correlations between the severity of depression and *SLC25A26* or *GAR1*, indicating that either their contributions are influenced by other factors or that their expression does not change in a consistent manner with the intensity of symptoms. Thus, these results highlight the role of gene expression patterns that can be used to stratify patients, anticipate the course of the disease, and create individualized treatment plans when combined with clinical severity evaluations like the PHQ-9 and HAM-D. To further clarify these associations and create reliable biomarker panels for clinical use, larger, severity-stratified cohorts are needed in future studies.

## 4. Discussion

Differential gene expression plays a major role in understanding the biological aspects of depression and related mood disorders. Several studies have demonstrated the importance of differential gene expression from various tissues of both the postmortem brain and the peripheral blood, revealing the complex and sex-specific patterns of modifications [35]. This study aimed to determine the potential role of blood-based biomarker genes by evaluating the differential expression of the selected five candidate genes, *GAR1*, *PER3*, *MTPAP*, *SLC25A26*, and *CD19*, in whole blood samples of depressive patients when compared with the healthy controls. These five genes were chosen based on their reported roles in ribosomal RNA processing (GAR1), mitochondrial metabolism (MTPAP, SLC25A26), circadian rhythm regulation (PER3), and immunological regulation (CD19). The quantitative gene expression profiling of these five candidate genes showed differential expression throughout the gene panel, with GAR1 and SLC25A26 showing mild, non-significant changes and CD19, MTPAP, and PER3 revealing significant changes. Receiver operating characteristics analysis (ROC) and pathway identification were also performed, along with the gene expression profiling of these candidate genes, to show the potential link between the molecular markers and the pathophysiology of depression. The combined five-gene model produced an AUC of 0.8850, according to receiver operating characteristic (ROC) analysis, demonstrating a strong discriminatory capacity between MDD cases and controls, whereas individual gene performance varied significantly.

The differential expression of all the five candidate genes highlighted the significant alteration in expression for CD19 in depression. The surface membrane antigen CD19 is mostly present in B-lymphocytes and is crucial for B-cell development and function, as well as immunological dysregulation [34].

CD19 is a gene that is significantly expressed among all the other genes that distinguishes suicide completers (MDD-S) from MDD-NS non-suicide deaths [35,36]. Previous studies have demonstrated the differential expression of CD19 in depression, specifically in patients who die by suicide [37]. They have also shown a pattern of up-regulation in blood tissue and downregulation in the dorsolateral prefrontal cortex (DLPFC). The idea that the major depressive disorder involves complex, region-specific molecular changes is supported by this tissue-specific divergence. Along with the elevated expressions of other markers such as CD6 and NR3C1,the upregulation of CD19 observed in peripheral blood in MDD-S patients has been explained as a component of an overactive immune response. Despite CD19 upregulation, several studies have found no significant differences in CD19+ B-cell counts between healthy individuals and people suffering from depression. This highlights that the chances of modifications at the transcript level do not always translate into changes in the cell populations’ phenotype. The current findings, however,should be considered as demonstrating general immunological dysregulation in depression, rather than any mechanism particular to suicidality, as the study did not classify subjects based on suicidal behavior [38,39].

Hence, all these findings support the pathophysiology of depression by emphasizing the role of inflammation and immunological dysregulation, and thus the observed pattern of CD19 underscores the potential role of the peripheral biomarker, particularly in severe or treatment-resistant patients with depressio [40].

In this study, MTPAP was considerably upregulated, with a fold change of 3.99, and *p* < 0.000001, in depression when compared to the healthy control, suggesting its potential and possible role in the pathophysiology of depression. MTPAP (Mitochondrial Poly(A) Polymerase), a mitochondrial enzyme, is responsible for the polyadenylation of the 3′ ends of mitochondrial mRNAs, a procedure that is necessary for transcript stability and effective mitochondrial protein synthesis. Since the gene is essential for preserving the integrity of mitochondrial mRNA, changes in MTPAP expression may hinder the production of mitochondrial proteins, interfere with oxidative phosphorylation, and exacerbate the deficiencies in energy metabolism that are frequently seen in MDD [41]. Thus, oxidative stress and inflammatory reactions have been frequently linked to mitochondrial malfunction, which is also known to result in depressive symptomatology [5]. These results suggest a potential association of mitochondrial homeostasis in depression, and MTPAP may serve as a potential biomarker of mitochondrial involvement in mood disorders.

A core circadian clock gene, PER3, demonstrated a differential expression that was consistent with previous evidence linking circadian rhythm disruption with mood, sleep habits, and biological rhythm regulation. PER3’s genetic variant or altered expression has been linked to sleep architecture disruptions and heightened susceptibility to bipolar illness and depression. By showing that PER3 VNTR genotypes affect the age of onset in bipolar I disorder, Barlattani et al. (2024) [24] interestingly illustrated how circadian gene variability may affect clinical phenomenology and illness trajectory. Previous animal studies have demonstrated that mitochondrial dysfunction caused by PER3 deficiency results in depression-like behaviors, including decreased sucrose preference and increased behavioral despair. In terms of mechanism, PER3 controls the NAMPT-NAD^+^-SIRT3 axis to govern mitochondrial energy metabolism. The pathophysiology of MDD is characterized by oxidative stress and mitochondrial dysfunction, which are exacerbated by its absence. This results in decreased ATP synthesis, decreased NAD^+^ levels, and impaired tricarboxylic acid cycle enzyme activity. According to intriguing human postmortem research, PER3 has been upregulated in the peripheral blood and dorsolateral prefrontal cortex (DLPFC) tissues of MDD patients who died by suicide. This suggests that severe depression may have pathoadaptive or compensatory responses that are specific to certain tissues. This discrepancy between the evidence from humans and animal studies could be due to the different regulatory networks that are active in genetic knockouts vs. naturally occurring mood disorders, or it could be the result of complicated temporal dynamics of PER3 expression in response to chronic stress. The PER3 upregulation observed in our study could be a compensating reaction to depression’s circadian and mitochondrial stress. According to previous studies connecting PER3 dysfunction to disturbed bioenergetic and circadian regulation in mood disorders, altered PER3 expression may have an impact on cellular energy balance and mitochondrial integrity. PER3 has the ability to influence mitochondrial metabolism and oxidative resilience through its involvement in the SIRT3 signaling pathway, which is dependent on NAD. Although these results point to a possible connection between circadian rhythm dysregulation and mitochondrial disruption in MDD, more functional validation and long-term research are needed to ascertain whether changes in PER3 expression are an adaptive reaction to long-term stress or a causal factor in disease mechanisms.

This study did not show any significantly differential expression of *SLC25A26*, a mitochondrial transporter for S-adenosylmethionine (SAM), between the patients with depressed and healthy controls. However, previous research has shown that *SLC25A26* is one of the most deferentially expressed genes in patients with major depressive disorder. Thus, SLC25A26 might be a peripheral biomarker linked to the most severe forms of depression, particularly those that are associated with an increased risk of suicide. The mitochondrial carrier protein that SLC25A26 encodes is functionally responsible for the transportation of S-adenosylmethionine (SAM) into the mitochondria. Maintaining the integrity, function, and regulation of gene expression of mitochondrial RNA depends on the transport of SAM, a crucial methyl donor involved in mitochondrial RNA methylation. Any disturbance to this transport mechanism may threaten the mitochondrial integrity and RNA processing, which could damage cellular metabolism and make people more susceptible to mental illnesses linked to stress. Interestingly, blood has some of the greatest levels of RNA methylation, despite having very low expression of SLC25A26. This suggests that even slight disruption of SAM transport may have disproportionate impacts on blood-based cellular functions. The biological function of SLC25A26 is consistent with more comprehensive data that links epigenetic modifications and mitochondrial dysfunction to the pathophysiology of depression, even if our results did not show a statistically significant change in expres on [15]. Maladaptive cellular reactions in MDD may be influenced by mitochondrial methylation disruption and altered SAM availability. Therefore, further investigation into *SLC25A26* and how it interacts with methylation pathways may offer a more profound understanding of the molecular processes behind depression, especially in high-risk groups.

In addition, this study demonstrated a modest and non-significant upregulation of *GAR1* in depressed patients compared to the healthy controls (fold change: 1.25, *p* = 0.470). This result implies that changes in GAR1 expression are not statistically significant in this group and should be regarded with caution. In order to digest and pseudouridylate ribosomal RNA and support ribosome synthesis and translational control, GAR1 encodes a tiny nucleolar ribonucleoprotein component. Even slight transcriptional variation may be a reflection of systemic effects of stress, altered protein synthesis, or immune–nucleolar signaling, all of which have been linked to mood disorders due to its essential biological role. The peripheral blood of people with major depressive disorder has been shown to have GAR1 dysregulation in previous transcriptome studies, including some that found differential expression in suicide completers. These results, however, may be context-specific or postmortem alterations, rather than reliable indicators of depression, because they were obtained from separate datasets. The current research suggests that GAR1 is unlikely to function as a significant standalone biomarker for depression in this group, due to the lack of statistical significance and the lack of enrichment in STRING-based functional annotation. However, GAR1’s inclusion in the combined multi-gene ROC panel only slightly improved the overall discriminative ability (AUC = 0.8850), indicating that when compared to mitochondrial, immune, and circadian genes, ribosomal and RNA-processing pathways may still be secondary in the larger molecular landscape of depression.

Overall, GAR1’s diagnostic use is still restricted in the absence of a notable expression variations, even though it may represent underlying cellular stress or translational dysregulation in depressed conditions. To determine if GAR1 indirectly contributes to the molecular processes of major depressive disorder, future research including larger, multi-center cohorts and functional validation at the protein and ribosome-biogenesis levels is necessary.

An AUC of 0.8850 was obtained from the combined ROC curve of all of the five genes, which is significant since it shows good discriminative power between depression cases and controls. This highlights the potential benefits of utilizing a gene expression panel, as opposed to depending solely on individual indicators. Such integrative biomarker techniques are justified by the multifactorial nature of depression, which involves immunological, metabolic, and neuronal dysfunction.

Hence, this study demonstrated that the patients with depression exhibit an altered expression of genes related to ribosomal biology, mitochondrial function, immunological response, and circadian control. These genes may be useful biomarkers or treatment targets for depression, as indicated by the considerable up-regulation of *CD19*, *PER3*, and *MTPAP*, as well as the good combined ROC performance. To validate these results and investigate molecular connections to depressive illness, more extensive research with a wider range of population and functional validation is necessary.

## 5. Conclusions

This study involved the identification of the gene expression levels of five selected gene candidates—*GAR1*, *PER3*, *MTPAP*, *SLC25A26*, and *CD19*—in newly diagnosed drug-naive patients with depression and compared those with the healthy controls, using the whole blood samples. The significant upregulation of *CD19*, *MTPAP*, and *PER3* among the five genes raises the possibility of their potential involvement in immune, mitochondrial and circadian regulatory mechanisms causing depression. SLC25A26 did not exhibit differential expression, but *GAR1* displayed modest non-significant elevation. Notably, *CD19* showed the greatest statistical significance and fold change, indicating a significant role for immune system dysregulation in the pathophysiology of depression. According to ROC analysis, a combined gene panel reveals a discriminating ability in distinguishing patients from controls, even if individual gene performance can be low (AUC = 0.8850, *p* < 0.0001), highlighting the possible diagnostic value of integrating multiple molecular markers. Network enrichment and co-expression analyses failed to identify the statistical significance in inter-gene interactions, and pathway insights indicated the possible involvement of immune dysregulation, mitochondrial dysfunction, disruption of the circadian rhythm, and translational control: mechanisms that are increasingly identified in depressive disorders.

### 5.1. Limitations

This study was a case–control study design and used a single-center sample collecting method, which may limit the generalizability of findings. Gene expression was only assessed at the mRNA level, and did not include protein-level validation or functional mechanistic studies. Even though oxidative stress markers and inflammatory cytokines have a mechanistic connection to immune and mitochondrial genes, they were not evaluated. Confounding factors such as medication status, lifestyle, or environmental influences were not controlled. Even though the sample size was sufficient for exploratory analysis, it is necessary to validate these findings in a larger and more diverse population to represent the heterogeneity.

### 5.2. Future Directions

To strengthen these findings, future research needs to focus on validating this gene panel in larger, multi-center cohorts and include longitudinal designs to monitor gene expression across treatment and recovery. A more comprehensive knowledge of the molecular foundations of depression might be made possible by integrating the transcriptomic data with proteomics, metabolomics, and clinical phenotyping. Functional assays and pathway-level validations are critical to clarify the mechanistic roles of these five genes in the pathophysiology of depression and to evaluate their translational potential as therapeutic targets or prognostic markers.

## Figures and Tables

**Table 1:** List of forward and reverse primers.

Gene	Forward Primer	Reverse Primer	Product Length
*MTPA*	TACTCAGGTGGCTTACGCTG	TAGCATTTCCCTACCACGGC	145 bp
*CD19*	CTAGCCTTCACCTACTGCCC	GGGCACCCAGCATGTAACT	117 bp
*PER3*	GGCAGATGATGTCCAAGCCTTAC	ATCGCCGTGGACAGGATCTGTG	353 bp
*GAR1*	GTCTTTTCGAGGCGGAGGTC	GGTCCTTGGTCTTGGCCTTT	195 bp
*SLC25A26*	CCGGGGGTATACTACGGTCA	GTGGGCTCTAGAGGGGGTAG	148 bp
*β-actin*	CATGTACGTTGCTATCCA	CTCCTTAATGTCACGCAC	250 bp

**Table 2:** Demographic characteristics among the case and the control group.

Parameter	Depression Group (*n* = 100)	Control Group (*n* = 100)	*p*-Value
Age (years, mean ± SD)	32.7 ± 7.3	32.3 ± 8.3	0.78
Gender, *n* (%)			
Male	57 (57%)	55 (55%)	0.76
Female	43 (43%)	45 (45%)	
PHQ-9 score, mean ± SD	20.3 ± 3.8	3.1 ± 1.2	<0.001
HAM-D score, mean ± SD	25.7 ± 4.2	2.9 ± 1.0	<0.001

**Table 3:** Blood-based differentially expressed genes between MDD patients and control group, fold change in MDD group relative to control group.

Gene	Mean (Control)	Mean (Case)	Fold Change	FC Direction	AUC	Interpretation
*PER3*	1.0296	−0.2476	2.42	UP	0.5150	Circadian involvement
*MTPAP*	−2.1146	−4.111	3.99	UP	0.9125	Mitochondrial link
*CD19*	−1.5964	−4.2176	6.15	UP	0.8613	Strong biomarker
*SLC25A26*	−4.0319	−4.3393	1.24	UP	0.9575	Not significant
*GAR1*	0.8853	0.5574	1.26	UP	0.5875	Modest marker

**Table 4:** Diagnostic performance metrics for each gene and the combined gene model.

Gene	AUC (95% CI)	Optimal Cut-Off (ΔCt)	Sensitivity (%)	Specificity (%)	Cross-Validated AUC (Mean ± SD)	Interpretation
*CD19*	0.8613 (0.82–0.90)	−3.79	100.0	71.5	0.852 ± 0.025	Excellent discrimination; immune-related marker
*MTPAP*	0.9125 (0.88–0.94)	−5.92	100.0	68.0	0.905 ± 0.021	Excellent accuracy; mitochondrial dysfunction signature
*PER3*	0.5150 (0.45–0.57)	−3.86	100.0	42.5	0.509 ± 0.028	Poor individual performance; circadian involvement only
*SLC25A26*	0.9575 (0.93–0.98)	−4.33	98.1	91.2	0.951 ± 0.017	Outstanding discrimination; methylation/mitochondrial biomarker
*GAR1*	0.5875 (0.52–0.65)	−4.17	70.0	55.0	0.580 ± 0.031	Modest discrimination; ribosomal marker
Combined Panel (5 genes)	0.8850 (0.85–0.92)	−3.33	97.5	82.0	0.876 ± 0.019	Excellent overall performance; multi-gene synergy

## Data Availability

The datasets generated and analyzed during the current study are available from **Public in Array Express** under the accession number **E-MTAB-15757** [31].

## Supplementary Materials

The following supporting information can be downloaded at: https://www.mdpi.com/article/10.3390/diagnostics16060884/s1, STROBE and STARD-2015-Checklist.

## References

[1] World Health Organization (2017). Depression and Other Common Mental Disorders: Global Health Estimates.

[2] Greenberg P.E., Fournier A.-A., Sisitsky T., Simes M., Berman R., Koenigsberg S.H., Kessler R.C. (2021). The Economic Burden of Adults with Major Depressive Disorder in the United States (2010 and 2018). PharmacoEconomics.

[3] Lang U.E., Walter M. (2017). Depression in the context of medical disorders: New Pharmacological pathways revisited. NeuroSignals.

[4] Liu Q., He H., Yang J., Feng X., Zhao F., Lyu J. (2020). Changes in the global burden of depression from 1990 to 2017: Findings from the Global Burden of Disease study. J. Psychiatr. Res..

[5] Uher R., Perlis R.H., Henigsberg N., Zobel A., Rietschel M., Mors O., Hauser J., Dernovsek M.Z., Souery D., Bajs M. (2012). Depression symptom dimensions as predictors of antidepressant treatment outcome: Replicable evidence for interest-activity symptoms. Psychol. Med..

[6] Willour V.L., Seifuddin F., Mahon P.B., Jancic D., Pirooznia M., Steele J., Schweizer B., Goes F.S., Mondimore F.M., MacKinnon D.F. (2012). A genome-wide association study of attempted suicide. Mol. Psychiatry.

[7] Malhi G.S., Mann J.J. (2018). Depression. Lancet.

[8] Rush A.J., Trivedi M.H., Wisniewski S.R., Nierenberg A.A., Stewart J.W., Warden D., Niederehe G., Thase M.E., Lavori P.W., Lebowitz B.D. (2006). Acute and Longer-Term Outcomes in Depressed Outpatients Requiring One or Several Treatment Steps: A STAR*D Report. Am. J. Psychiatry.

[9] Targum S.D., Schappi J., Koutsouris A., Bhaumik R., Rapaport M.H., Rasgon N., Rasenick M.M. (2022). A novel peripheral biomarker for depression and antidepressant response. Mol. Psychiatry.

[10] Otte C., Gold S.M., Penninx B.W., Pariante C.M., Etkin A., Fava M., Mohr D.C., Schatzberg A.F. (2016). Major depressive disorder. Nat. Rev. Dis. Primers.

[11] Bi Y., Ren D., Guo Z., Ma G., Xu F., Chen Z., An L., Zhang N., Ji L., Yuan F. (2021). Influence and interaction of genetic, cognitive, neuroendocrine and personalistic markers to antidepressant response in Chinese patients with major depression. Prog. Neuro-Psychopharmacol. Biol. Psychiatry.

[12] Fanelli G., Benedetti F., Wang S.-M., Lee S.-J., Jun T.-Y., Masand P.S., Patkar A.A., Han C., Serretti A., Pae C.-U. (2020). Reduced plasma Fetuin-A is a promising biomarker of depression in the elderly. Eur. Arch. Psychiatry Clin. Neurosci..

[13] Redei E.E., Andrus B.M., Kwasny M.J., Seok J., Cai X., Ho J., Mohr D.C. (2014). Blood transcriptomic biomarkers in adult primary care patients with major depressive disorder undergoing cognitive behavioral therapy. Transl. Psychiatry.

[14] Maes M. (1992). A significantly increased number and percentage of B cells in depressed subjects: Results of flow cytometric measurements. J. Affect. Disord..

[15] Nagy C., Suderman M., Yang J., Szyf M., Mechawar N., Ernst C., Turecki G. (2015). Astrocytic abnormalities and global DNA methylation patterns in depression and suicide. Mol. Psychiatry.

[16] Stolfi F., Brasso C., Raineri D., Landra V., Mazzucca C.B., Ghazanfar A., Scotti L., Sinella R., Villari V., Cappellano G. (2025). Deep immunophenotyping of circulating immune cells in major depressive disorder patients reveals immune correlates of clinical course and treatment response. Brain Behav. Immun. Health.

[17] Su L., Shuai Y., Mou S., Shen Y., Shen X., Shen Z., Zhang X. (2022). Development and validation of a nomogram based on lymphocyte subsets to distinguish bipolar depression from major depressive disorder. Front. Psychiatry.

[18] Weckmann K., Labermaier C., Asara J.M., Müller M.B., Turck C.W. (2014). Time-dependent metabolomic profiling of Ketamine drug action reveals hippocampal pathway alterations and biomarker candidates. Transl. Psychiatry.

[19] Levey D.F., Polimanti R., Cheng Z., Zhou H., Nuñez Y.Z., Jain S., He F., Sun X., Ursano R.J., Kessler R.C. (2019). Genetic associations with suicide attempt severity and genetic overlap with major depression. Transl. Psychiatry.

[20] Artioli P., Lorenzi C., Pirovano A., Serretti A., Benedetti F., Catalano M., Smeraldi E. (2007). How do genes exert their role? Period 3 gene variants and possible influences on mood disorder phenotypes. Eur. Neuropsychopharmacol..

[21] Babenko V.N., Smagin D.A., Galyamina A.G., Kovalenko I.L., Kudryavtseva N.N. (2018). Altered Slc25 family gene expression as markers of mitochondrial dysfunction in brain regions under experimental mixed anxiety/depression-like disorder. BMC Neurosci..

[22] Bunney B.G., Li J.Z., Walsh D.M., Stein R., Vawter M.P., Cartagena P., Barchas J.D., Schatzberg A.F., Myers R.M., Watson S.J. (2015). Circadian dysregulation of clock genes: Clues to rapid treatments in major depressive disorder. Mol. Psychiatry.

[23] do Sacramento P.M., Sales M., Kasahara T.d.M., Monteiro C., Oyamada H., Dias A.S.O., Lopes L., Castro C.T., Rossi Á.D., Milioni L.M. (2022). Major depression favors the expansion of Th17-like cells and decrease the proportion of CD39+Treg cell subsets in response to myelin antigen in multiple sclerosis patients. Cell. Mol. Life Sci..

[24] Barlattani T., Soltmann B., D’Amelio C., Socci V., Pacitti F., Pompili M., Ritter P. (2024). The influence of PER3 VNTR genotypes on the age of onset in a group of bipolar I disorder patients: An exploratory study. Int. J. Bipolar Disord..

[25] Sun Y., Fu Z., Bo Q., Mao Z., Ma X., Wang C. (2020). The reliability and validity of PHQ-9 in patients with major depressive disorder in psychiatric hospital. BMC Psychiatry.

[26] Fiori L.M., Turecki G. (2010). Gene expression profiling of suicide completers. Eur. Psychiatry.

[27] Clough E., Barrett T. (2016). The Gene Expression Omnibus Database. Methods Mol. Biol..

[28] Patrone D., Alessio N., Antonucci N., Brigida A.L., Peluso G., Galderisi U., Siniscalco D. (2022). Optimization of Peripheral Blood Mononuclear Cell Extraction from Small Volume of Blood Samples: Potential Implications for Children-Related Diseases. Methods Protoc..

[29] Venkatachalapathy Y., Suresh P.K.K., Balraj T.H., Venkatesan V., Geminiganesan S., C D M.P. (2024). Clinico-demographic and biochemical correlation of inflammatory gene expression in pediatric nephrotic syndrome. Mol. Biol. Rep..

[30] Unal I. (2017). Defining an Optimal Cut-Point Value in ROC Analysis: An Alternative Approach. Comput. Math. Methods Med..

[31] Montenegro J.D. (2022). Gene Co-expression Network Analysis. Methods Mol. Biol..

[32] Reimand J., Isserlin R., Voisin V., Kucera M., Tannus-Lopes C., Rostamianfar A., Wadi L., Meyer M., Wong J., Xu C. (2019). Pathway enrichment analysis and visualization of omics data using g:Profiler, GSEA, Cytoscape and EnrichmentMap. Nat. Protoc..

[33] Shannon P., Markiel A., Ozier O., Baliga N.S., Wang J.T., Ramage D., Amin N., Schwikowski B., Ideker T. (2003). Cytoscape: A Software Environment for Integrated Models of Biomolecular Interaction Networks. Genome Res..

[34] Wang K., Wei G., Liu D. (2012). CD19: A biomarker for B cell development, lymphoma diagnosis and therapy. Exp. Hematol. Oncol..

[35] Mamdani F., Weber M.D., Bunney B., Burke K., Cartagena P., Walsh D., Lee F.S., Barchas J., Schatzberg A.F., Myers R.M. (2022). Identification of potential blood biomarkers associated with suicide in major depressive disorder. Transl. Psychiatry.

[36] Kohli M.A., Lucae S., Saemann P.G., Schmidt M.V., Demirkan A., Hek K., Czamara D., Alexander M., Salyakina D., Ripke S. (2011). The neuronal transporter gene SLC6A15 confers risk to major depression. Neuron.

[37] Zhou B., Wu T., Li H., Yang J., Ma Z., Ling Y., Ma H., Huang C. (2024). Identification of CD19 as a shared biomarker via PPARγ/β-catenin/Wnt3a pathway linking psoriasis and major depressive disorder. J. Affect. Disord..

[38] Le-Niculescu H., Levey D.F., Ayalew M., Palmer L., Gavrin L.M., Jain N., Winiger E., Bhosrekar S., Shankar G., Radel M. (2013). Discovery and validation of blood biomarkers for suicidality. Mol. Psychiatry.

[39] Levey D.F., Niculescu E.M., Le-Niculescu H., Dainton H.L., Phalen P.L., Ladd T.B., Weber H., Belanger E., Graham D.L., Khan F.N. (2016). Towards understanding and predicting suicidality in women: Biomarkers and clinical risk assessment. Mol. Psychiatry.

[40] Dowlati Y., Herrmann N., Swardfager W., Liu H., Sham L., Reim E.K., Lanctôt K.L. (2010). A Meta-Analysis of Cytokines in Major Depression. Biol. Psychiatry.

[41] Reis P.P., Waldron L., Goswami R.S., Xu W., Xuan Y., Perez-Ordonez B., Gullane P., Irish J., Jurisica I., Kamel-Reid S. (2011). mRNA transcript quantification in archival samples using multiplexed, color-coded probes. BMC Biotechnol..

